# Diseased Meats and Foul Water, Considered as Causes of Disease

**Published:** 1880-06

**Authors:** Henry M. Lyman

**Affiliations:** Professor of Physiology, Etc., Rush Medical College, Chicago


### ARTICLE II.

### DISEASED MEATS AND FOUL WATER CONSIDERED AS CAUSES OF DISEASE. By HENRY M. LYMAN, M.D., Professor of Physiology, Etc., Rush Medical College, Chicago.

In view of the peremptory manner in which our newspaper oracles assign to this or to that article of food and drink the cause of the most varied diseases, it seems desirable to ask whether any exact knowledge may exist regarding the relations between our aliments and the maladies which afflict the community. Scarlet fever, diphtheria, malarial fevers, etc., etc., have recently been referred to contamination of our water-supply. The sanitary authorities consider "emaciated beef, sour hams, cholera hogs," and various other kinds of game, so dangerous to the public health that they are daily presenting the fertilizer-factories with hundreds of pounds of meat, of which no inconsiderable portion might otherwise find its way to the mouths of certain classes of our population. How far this course may be necessary and justifiable becomes, therefore, an important question. In his recent work on Epidemic Diseases, Professor Léon Colin has carefully reviewed the whole subject, and has arrived at the conclusions which I shall present in the following abstract of his chapter on Food and Water, considered as causes of disease.

If animal food, says our author, can be considered the cause of epidemic disease, it is through its absence rather than through its quality. The cattle-plague itself does not vitiate the character of beef furnished by cattle dying of the disease. The principal danger to man in such cases lies in the risks of a meat famine caused by the general destruction of the herds. The experience of Chauveau might lead to a belief in the danger of consuming the flesh of animals affected with diseases communicable to the human species, but experience proves the contrary. During the first French Revolution the poor of Saint Germain and of Alfort were preserved from starvation by the use of the flesh of glandered horses which they devoured without any injury whatever. The same thing is true in the case of meat derived from animals affected with anthrax. Flesh which contains the germs of trichina, of echinococcus, and similar parasites, is alone really dangerous as an article of food. It is the class of aliments derived from the vegetable kingdom which is the most likely to prove injurious to the health of man. Scurvy, leprosy, pellagra, ergotism are familiar examples of alimentary diseases. Dysentery and diarrhœa have been charged against the abuse of fruits; but these affections are usually produced by causes which simply coincide, in point of time, with the ripening of fruit. The absence of food, such as occurs in famine, often profoundly modifies nutrition without the production of any of the maladies which are properly associated with defective or vicious alimentation. Under such circumstances, the results appear as an aggravation of the majority of the prevailing diseases, and an increase of the mortality, which rises as the famine becomes intensified. Thus we see typhus fever, relapsing fever, dysentery and similar diseases associated with a deficient alimentation. The intimate relation which exists between the rate of mortality and the price of provisions has long been remarked. High prices mean scarcity among the poor; and a scanty food supply soon effects an increased predisposition to disease. The results of such varying conditions are fully illustrated on a large scale in the Asiatic empires. In districts where the ancient irrigation works have been restored, so as to furnish abundance of water for the crops, cholera and other wasting diseases cease to prevail; the population rapidly increases, and good health becomes universal. The first care of our sanitary authorities, when they undertake to deal with the food-supply of the people, should be directed toward measures tending to increase its quantity and to decrease its expense. Great caution, therefore, should be exercised in the matter of confiscating and destroying edible meats. Such destruction of a portion of the supply enhances the cost of the remainder and reduces the quantity available for the nourishment of the poor. It is only necessary to stand for a short time in any frequented market, to see how great is the number of unfortunate creatures who are compelled to approach the butcher with the humble inquiry for cheap meat. The world is full of people who would gladly feast upon the refuse which the rich consider hardly fit for a dog. If it can be shown that much of the meat which is now subject to destruction in our market is really harmless as food, its loss is a tremendous waste of capital in the form of human health and vigor.

I am well aware that the attempt has recently been made to show that typhoid fever may be caused by the consumption of tainted meats. A careful analysis, however, shows that the cases quoted as cases of typhoid fever are really cases of intestinal irritation produced by the use of meats in a state of actual putrefaction. This only occurs among those who use compressed meats, old sausages, putrid cheese, etc.; and the cases recorded bear no kind of relation with the use of such articles. They belong to the category of cases of poisoning by the eating of custards—articles of diet which are ordinarily perfectly harmless but which may occasionally be transformed into deadly poisons, probably by the growth of poisonous fungi or algæ in their substance. In the *British Medical Journal* for March 20, 1880, may be found a collection of such cases; but of them all it may well be remarked, in the language of Colin (p. 147), that such examples are ensphered in a multitude of cases where "similar diet has often been used with an impunity which forbids its enumeration among the causes of typhoid fever."

*Drinking Water.*—A certain class of medical writers are accustomed to accuse the water supply of a community as the vehicle of communication by which the efficient causes of malarial fever, cholera, typhoid fever, etc., etc., are conveyed into the system. This doctrine is, just now, exceedingly fashionable among English authors, who seem ambitious to surpass each other in the display of uncritical credulity. Without denying the communication of parasitic diseases by the injection of water containing the eggs of worms, our author examines the evidence advanced in behalf of the communication of the various epidemic diseases and arrives at the following conclusions:

I. *Intermittent fevers* are caused by respirable malaria. Numerous examples are given in illustration of the fact that people who drink so-called malarious waters, but who do not breathe the atmosphere of the malarious locality, are not attacked with malarial fevers; while, on the contrary, people who, like the Romans, dwell in a malarious atmosphere, but who, at the same time, drink the pure aqueduct water from the healthy Appennines, are scourged with fever. Swamp waters have been considered causative of fever because, in the vast majority of cases, the people who drink them are at the same time breathing the malarious atmosphere which is associated with them. In this connection it is interesting to note that in the recent experiments of Professor Klebs at Rome (*Med. Times and Gazette*, Jan. 17, 1880) the injection of rabbits "with water standing over the marshy ground remained without effect," while infusion of the organic matter in the soil produced grave febrile disturbance.

II. *Typhoid Fever.* English and German authors have collected a number of examples supposed to prove the communication of typhoid fever by drinking water. The story of the Lausen epidemic is perhaps the most extraordinary example of such narratives. It is given in full by Dr. Cayley, in a recent lecture on typhoid fever (*Brit. Med. Journ.*, March 13, 1880).

Ballard and others have attempted to make out a similar method of water contamination as the cause of local epidemics of typhoid fever. But when, as in the Lausen epidemic, it has been shown that the disease had been prevailing less than two miles from the village for nearly two months previous to the greater outbreak, it would seem that all the credulity of an homœopathist was needed in order to adopt the hypothesis that the fever poison was conveyed by filtration through a mountain a mile thick, rather than the more reasonable view that it had found its way over or around the obstacle by the vehicle of personal intercourse. It, however, should not be forgotten that the use of water charged with decomposing organic matter may become one of the predisposing causes, if not an actual exciting cause of typhoid fever. This may especially be observed in localities where fecal accumulations are allowed to filter into adjacent wells. Ordinary excrementitious substances may then help to originate the fever.

III. *Cholera.*—The propagation of cholera by contiguity, its rapid diffusion, and its dispersion without regard to community of water-supply, all seem to negative the idea of its propagation through the medium of drinking water. When cholera was raging in Paris it did not prevail in the towns which took their water from the Seine below the city. The oft quoted examples of local prevalence of the disease among people using a common well or spring, with cessation of the epidemic when the water-supply was changed, may be explained by reference to the well-known brevity of local epidemics. While it may be admitted that water charged with putrefying organic matter may disorder the general health, thus undermining the power of resisting disease, it cannot be considered a direct cause of cholera.

IV. *Dysentery and Diarrhœa.*—These are the maladies in the production of which foul water is most efficacious. Examples without number exhibit the relation between stagnant waters, filled with decaying matter, and these intestinal diseases.

V. As for *scarlet-fever*, *diphtheria* and all other similar diseases, there is not the slightest evidence that they have any direct connection with the character of the water-supply. Even the attempt to connect the occurrence of ulcerative stomatitis with the use of melting snow as a drink has failed.

That water over-charged with decomposing organic matter is dangerous to health is universally admitted, and we have presented a view of the diseases which it may cause, as well as those which it can influence only in the most remote and indirect manner. It remains to inquire what degree of organic pollution (I have excluded from this essay all consideration of the dangers arising from the use of waters containing an excess of mineral matter) may be tolerated with impunity. The English Commission on River Pollution, proposed the following (Nature, Jan. 15, 1874, p. 197) as a standard beyond which organic matter could not be increased without danger: "Any liquid containing, *in solution*, more than two parts by weight of organic carbon, or 0.3 part by weight of organic nitrogen in 100,000 parts by weight." The test furnished by the French Academicians (Nature, June 10, 1875, p. 116) is sufficiently simple for popular use. The results of their investigations "seem to prove that water in which animals and plants of higher organizations will thrive is fit to drink; and, on the other hand, water in which only the infusioria and lower cryptograms will grow is unhealthy." Tried by either of these standards our Chicago water supply is still beyond suspicion.

The latest deliverance on this subject is from the pen of Prof. Tidy, who, on the 18th of March, 1880, read before the London Chemical Society, a paper on River-water. His conclusions regarding sewage will be read with interest by our friends along the Illinois river. His reporter says, (Nature, March 25, 1880, p. 507): "From inspection of the effect produced by sewage on rivers, from analyses of the river-waters, and from experiment, the author concludes that the oxidation of the organic matter of sewage takes place, when mixed with unpolluted water and allowed a certain flow, with extreme rapidity. The various methods of artificial purification are discussed; of these filtration through sand is preferred. Under the third category the arguments for and against the use of river water for drinking purposes are examined; it is shown that the death-rates of towns supplied by wells and of those supplied by rivers are practically alike, and that in London there is very little to choose, as regards mortality, between districts supplied with well-water and those supplied by river-water; and while admitting that, as a matter of sentiment, he would prefer well-water, the author contends that there is no reason for supposing that the *materies morbi*, whether it exists as a germ or not, can resist oxidation, which is efficient in destroying other organic matter, as proved by chemical analysis. The author finally submits the two following conclusions: 1. That when sewage is discharged into running water, provided the dilution with pure water be sufficient, the whole of it, after the run of a few miles, will be efficiently got rid of. 2. That facts indicate that whatever may be the actual cause of certain diseases, the *materies morbi* which finds its way into the river is destroyed along with the organic impurity."

